# Yeast Vps13 is Crucial for Peroxisome Expansion in Cells With Reduced Peroxisome-ER Contact Sites

**DOI:** 10.3389/fcell.2022.842285

**Published:** 2022-02-17

**Authors:** Wei Yuan, Arman Akşit, Rinse de Boer, Arjen M. Krikken, Ida J. van der Klei

**Affiliations:** Molecular Cell Biology, Groningen Biomolecular Sciences and Biotechnology Institute, University of Groningen, Groningen, Netherlands

**Keywords:** peroxisome, yeast, contact site, VPS13, endoplasmic reticulum

## Abstract

In the yeast *Hansenula polymorpha* the peroxisomal membrane protein Pex11 and three endoplasmic reticulum localized proteins of the Pex23 family (Pex23, Pex24 and Pex32) are involved in the formation of peroxisome-ER contact sites. Previous studies suggested that these contacts are involved in non-vesicular lipid transfer and important for expansion of the peroxisomal membrane. The absence of Pex32 results in a severe peroxisomal phenotype, while cells lacking Pex11, Pex23 or Pex24 show milder defects and still are capable to form peroxisomes and grow on methanol. We performed transposon mutagenesis on *H. polymorpha pex11* cells and selected mutants that lost the capacity to grow on methanol and are severely blocked in peroxisome formation. This strategy resulted in the identification of Vps13, a highly conserved contact site protein involved in bulk lipid transfer. Our data show that peroxisome formation and function is normal in cells of a *vps13* single deletion strain. However, Vps13 is essential for peroxisome biogenesis in *pex11.* Notably, Vps13 is also required for peroxisome formation in *pex23* and *pex24* cells. These data suggest that Vps13 is crucial for peroxisome formation in cells with reduced peroxisome-endoplasmic reticulum contact sites and plays a redundant function in lipid transfer from the ER to peroxisomes.

## Introduction

Peroxisomes are ubiquitous organelles. Their function and abundance continuously changes in response to cellular needs (Smith and Aitchison, 2013). During peroxisome growth, the organelles incorporate matrix and membrane proteins a well as membrane lipids. In mammals, membrane contact sites (MCSs) between peroxisomes and the endoplasmic reticulum (ER) function in non-vesicular transport of lipids from the ER to the peroxisomal membrane. These MCSs contain peroxisome bound members of the Acyl-CoA binding domain containing proteins (ACBDs), ACBD5 and ACBD4, and the ER-localized VAP proteins VAPA and VAPB. VAP proteins are highly conserved ER membrane proteins that play a role in various processes, including lipid transport (Lev et al., 2008). At mammalian peroxisome-ER contact sites ACBD5/ACBD4 interact through a FFAT-like motif with both VAP proteins (Costello et al., 2017; Hua et al., 2017; Islinger et al., 2020). Recent studies showed that human VPS13D, a bulk lipid transporter, is also important for peroxisome biogenesis and transport of lipids from the ER to peroxisomes (Baldwin et al., 2021; Guillen-Samander et al., 2021).

Studies in *Saccharomyces cerevisiae* revealed that also in this organism the peroxisomal membrane can receive membrane lipids via non-vesicular transport (Raychaudhuri and Prinz, 2008). These lipids may derive from various sources including the ER, the vacuole, the mitochondrion and the Golgi apparatus (Rosenberger et al., 2009; Flis et al., 2015). Also, in *S. cerevisiae* peroxisomes form contact sites with many other cellular membranes (Shai et al., 2016; Shai et al., 2018). However, proteins involved in non-vesicular lipid transport to yeast peroxisomes have not been identified yet. In the yeast *Hansenula polymorpha* peroxisomes can form various MCSs. Contacts have been described with the plasma membrane, the ER, mitochondria and vacuoles (Wu et al., 2019). ER-localized peroxins of the Pex23 family (Pex23, Pex24 and Pex32) together with the peroxisomal membrane protein (PMP) Pex11 play a role in the formation of peroxisome-ER contacts (Wu et al., 2020). Similarly, members of the *S. cerevisiae* Pex23 family (called Pex28, Pex29, Pex30, Pex31 and Pex32 (Jansen et al., 2021)) have been implicated in the formation of peroxisome ER contact sites (David et al., 2013; Mast et al., 2016). *S. cerevisiae* Pex23 family proteins also have been implicated in other processes, such as the regulation of pre-peroxisomal vesicle (PPV) formation from the ER (David et al., 2013; Joshi et al., 2016; Mast et al., 2016; Wang et al., 2018) and the biogenesis of lipid bodies (Joshi et al., 2018). Studies in *S. cerevisiae* suggested that Inp1, a protein essential for retention of peroxisomes in yeast mother cells, plays a role in the formation of peroxisome-ER contact sites (Knoblach et al., 2013). However, recent studies showed that Inp1 associates peroxisomes to the plasma membrane (Hulmes et al., 2020; Krikken et al., 2020).

The absence of *H. polymorpha* Pex23, Pex24 or Pex32 leads to reduction, but not a complete loss, of peroxisome-ER contacts. This reduction is accompanied by a decrease in the cellular peroxisomal membrane surface, suggesting that these peroxisome-ER contacts are important for lipid transfer.

The absence of the *H. polymorpha* Pex32 causes the most severe peroxisome-ER MCS defect, which is accompanied by mislocalization of a portion of the peroxisomal matrix proteins to the cytosol. As a consequence *pex32* cells are unable to grown on media containing methanol as sole carbon source (van der Klei et al., 2006). *pex23, pex24* and *pex11* cells show milder peroxisomal defects and still are capable to grow on methanol, although the doubling times are increased (Krikken et al., 2009; Wu et al., 2020).

We hypothesized that these weaker phenotypes are due to functional redundancy of proteins of the peroxisome-ER MCS. To identify these redundant proteins, we performed transposon mutagenesis of *H. polymorpha pex11* cells and selected mutants that fully lost the capacity to grow on methanol. This screen resulted in the identification of Vps13, a highly conserved protein that is responsible for lipid transport and localizes to multiple MCSs in eukaryotic cells. We show that like cells of the *pex11 vps13* double deletion strain, also *pex23 vps13* and *pex24 vps13* cells are unable to utilize methanol. In these double deletion strains very small peroxisomes still occur. PMPs are normally sorted to these organelles, but the bulk of the matrix proteins mislocalize to the cytosol. This suggests that peroxisomes can still form but are unable to grow and incorporate all matrix proteins. Cells of a single *vps13* deletion strain contain normal peroxisomes and grow on methanol like wild type control cells.

These data indicate that Vps13 is essential for peroxisome growth in cells that have reduced peroxisome-ER MCSs.

## Materials and Methods

### Strains and Growth Conditions

*The H. polymorpha* and *S. cerevisiae* strains used in this study are listed in the Supplementary Table S1and S2. *H. polymorpha* cells were grown in batch cultures at 37°C on mineral media (MM) (van Dijken et al., 1976) containing 0.5% glucose, 0.5% methanol or a mixture of 0.5% methanol and 0.05% glycerol (MM-M/G) as carbon sources and 0.25% ammonium sulfate as nitrogen sources. *S. cerevisiae* cells were grown at 30°C on media containing 0.5% glucose and 0.25% ammonium sulfate. When required, amino acids were added to the media to a final concentration of 30 μg/ml. *Escherichia coli* DH5α and DB3.1 were used for cloning.

### Plasmids and Molecular Techniques

GFP-SKL and DsRed-SKL are peroxisomal matrix markers appended with the peroxisomal targeting signal -SKL. In *H. polymorpha* the encoding genes were expressed under control of the *TEF1* or *AOX* promoter, in *S. cerevisiae* under control of the *MET25* promoter. For expression of *H. polymorpha* genes encoding various peroxisomal membrane proteins under control of their endogenous promoter, approximately 500 nucleotides upstream from the ORF were included. For the expression under control of a strong promoter the full length gene was cloned. All plasmids were linearized and integrated in the genome as described before (Faber et al., 1994). Plasmids used in this study are listed in Supplementary Table S3. All deletions were confirmed by Southern blotting. For DNA and amino acid sequence analysis, the Clone Manager 5 program (Scientific and Educational Software, Durham, NC) was used. Transposon mutagenesis of *H. polymorpha pex11* DsRed-SKL, isolation of total genomic DNA and sequencing of genomic insert was performed as described before (van Dijk et al., 2001).

DNA restriction enzymes were used as recommended by the suppliers (Thermo Scientific or New England Biolabs). Polymerase chain reactions (PCR) for cloning were carried out with Phusion High-Fidelity DNA Polymerase (Thermo Scientific). Colony PCR was carried out using Phire polymerase (Thermo Scientific). For DNA and amino acid sequence analysis, the Clone Manager 5 program (Scientific and Educational Software, Durham, NC) was used.

### Microscopy

Fluorescence microscopy images were captured using an Axio Scope A1 (Carl Zeiss) with a 100 × 1.30 NA Plan Neofluar objective, Micro-Manager 1.4 software and a Coolsnap HQ2 camera (Photometrics). GFP was visualized with a 470/40 nm band pass excitation filter, a 495 nm dichromatic mirror, and a 525/50 nm band-pass emission filter. mCherry was visualized with a 587/25 nm band pass excitation filter, a 605 nm dichromatic mirror, and a 647/70 nm band-pass emission filter. DsRed fluorescence was visualized with a 546/12 nm bandpass excitation filter, a 560 nm dichromatic mirror, and a 575–640 nm bandpass emission filter. Image analysis was carried out using ImageJ and Adobe Photoshop CS6 software.

To quantify peroxisomes random images of cells were taken as a stack using a confocal microscope (LSM510, Carl Zeiss) and photomultiplier tubes (Hamamatsu Photonics) and Zen 2009 software (Carl Zeiss). Z-Stack images were made containing 14 optical slices and the GFP signal was visualized by excitation with a 488 nm argon ion laser (Lasos), and a 500–550 nm bandpass emission filter. Peroxisomes were quantified using a custom made plugin for ImageJ (Thomas et al., 2015).

Electron microscopy was performed as described previously (Knoops et al., 2014). For morphological analysis cells were fixed in 1.5% potassium permanganate, post-stained with 0.5% uranyl acetate and embedded in Epon. Immuno-EM was performed as described previously (Knoops et al., 2014) using anti-Pex14 antibodies (Komori et al., 1997) followed by goat-anti-mouse antibodies conjugated to 6 nm gold (Aurion, Netherlands). Ultrathin sections were viewed in a Philips CM12 TEM.

## Results

### Vps13 is Required for Peroxisome Biogenesis in Yeast *pex11* Cells

**FIGURE 1:**
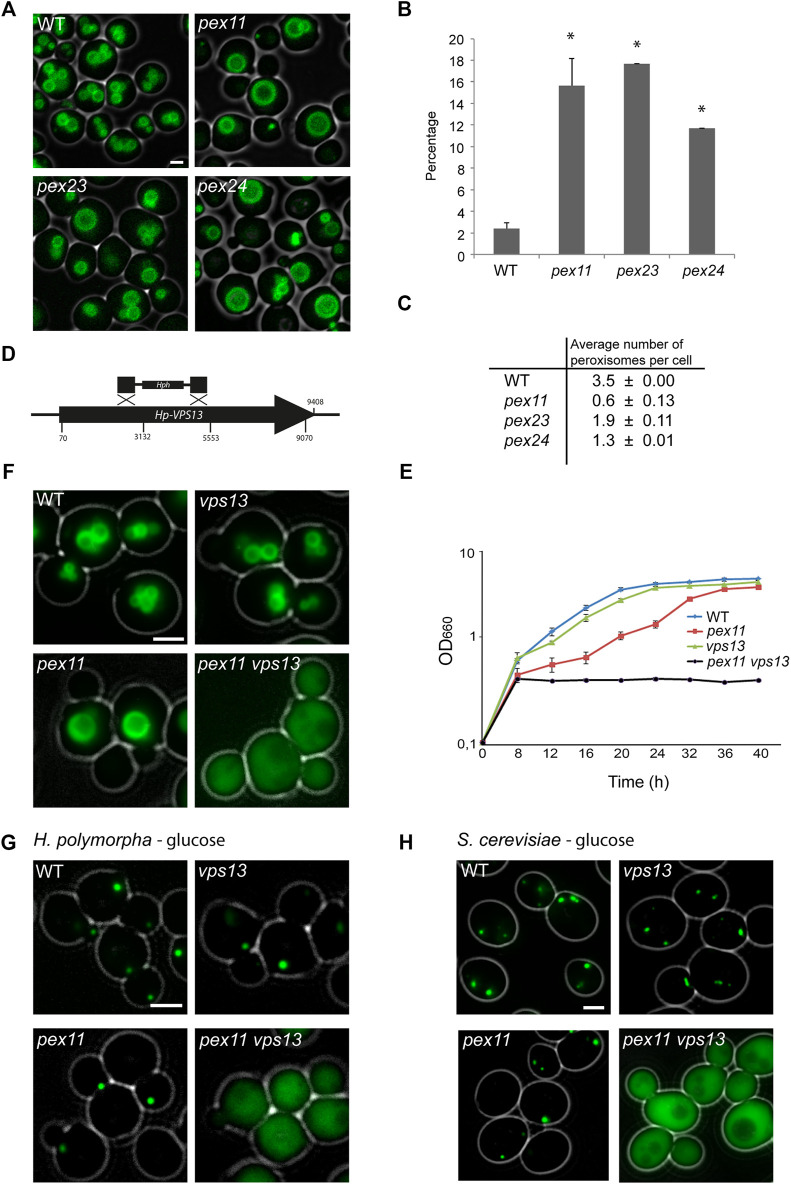
*VPS13* is required for peroxisome formation in *pex11* cells. *H. polymorpha* cells were grown for 16 h on methanol medium unless indicated otherwise. **(A)** FM analysis of the indicated *H. polymorpha* strains producing the peroxisomal membrane marker PMP47-GFP. Scale bar 2 µm. **(B)** Percentage of peroxisomes with a diameter >1 μm. 2 × 660 peroxisomes from two independent cultures were quantified. Two-tailed Student’s t test was performed. *, *p* < 0.05. *pex11*: 0.016, *pex23*: 0.003, *pex24*: 0.005. The error bars represent standard deviation (SD). **(C)** Average number of peroxisomes per cell (±SD) of the indicated strains. 2 × 660 cells from two independent cultures were quantified. **(D)** Schematic representation of the *H. polymorpha VPS13* gene, showing the four positions where transposon insertion occurred as well as the region that was replaced (nt 2430–5436) by Zeo^r^ to disrupt *VPS13*. **(E)** Growth curves of the indicated *H. polymorpha* strains on methanol medium. Error bars represent SD (*n* = 2). **(F)** FM images of the indicated *H. polymorpha* strains producing GFP-SKL. Cells were grown for 16 h on medium containing a mixture of methanol and glycerol. Scale bar: 2 µm. **(G)** FM images of the indicated strains grown on glucose medium (*pex11*.P_TEF_GFP-SKL, *vps13*P_TEF_GFP-SKL and *pex11 vps13*P_TEF_GFP-SKL). **(H)** FM analysis of the indicated *S. cerevisiae* strains producing GFP-SKL and grown on glucose. Scale bars: 2 μm.

Previously, we showed that like *H. polymorpha pex11*, *pex23* and *pex24* cells still contain peroxisomes (Krikken et al., 2009; Wu et al., 2020). However, peroxisome numbers are reduced accompanied by the occurrence of organelles with a relatively large diameter. We confirmed these observations by quantitative analysis of fluorescence microscopy (FM) images of these three deletion strains. The percentage of relatively large peroxisomes (diameter >1 µm) is significantly enhanced in *pex11, pex23* and *pex24* cells (Figures 1A,B). The lowest peroxisome numbers were observed in *pex11* cells (Figures 1A,C), which we selected to perform transposon mutagenesis.

Transformants were isolated that were still capable to grow on glucose, but not on methanol (Mut^−^), indicative for severe peroxisome biogenesis defects. FM analysis revealed that out of the 100 Mut^−^ strains obtained, 42 displayed mislocalization of the red fluorescent peroxisomal matrix marker DsRed-SKL. Sequencing of the genomic regions flanking the integrated pREMI-Z cassette resulted in the identification of 17 different genes (Supplementary Table S4). As expected these included various *PEX* genes, because mutations in most *PEX* genes result in a Mut^−^ phenotype due to mislocalization of matrix proteins. In 9 of the 17 identified mutants the transposon was integrated in *VPS13*. In four mutants the transposon was integrated at different positions in the *VPS13* open reading frame, whereas in the remaining five mutants deletions or truncations of the *VPS13* gene occurred (Figure 1D).

To validate this result, we constructed a *pex11 vps13* double mutant (Figure 1D). Cells of this strain, but not of the *pex11* or *vps13* single deletion strains, were unable to grow on methanol (Figure 1E). Moreover, peroxisomal matrix proteins were mislocalized to the cytosol of cells in the *pex11 vps13* double deletion strain, but not in *pex11* or *vps13* cells. This phenotype was observed both when cells were grown in peroxisome inducing media (methanol; Figure 1F) or peroxisome repressing media (glucose; Figure 1G).

In *S. cerevisiae* essentially the same observations were made: the peroxisome matrix marker GFP-SKL was properly imported into peroxisomes of *pex11* and *vps13* cells, but mislocalized to the cytosol in *pex11 vps13* double deletion cells (Figure 1H).

Summarizing, in *H. polymorpha* and *S. cerevisiae* deletion of *VPS13* in a *pex11* deletion strain strongly affects peroxisome biogenesis.

### *pex11 vps13* Cells Contain Small, Import Competent Peroxisomes

**FIGURE 2:**
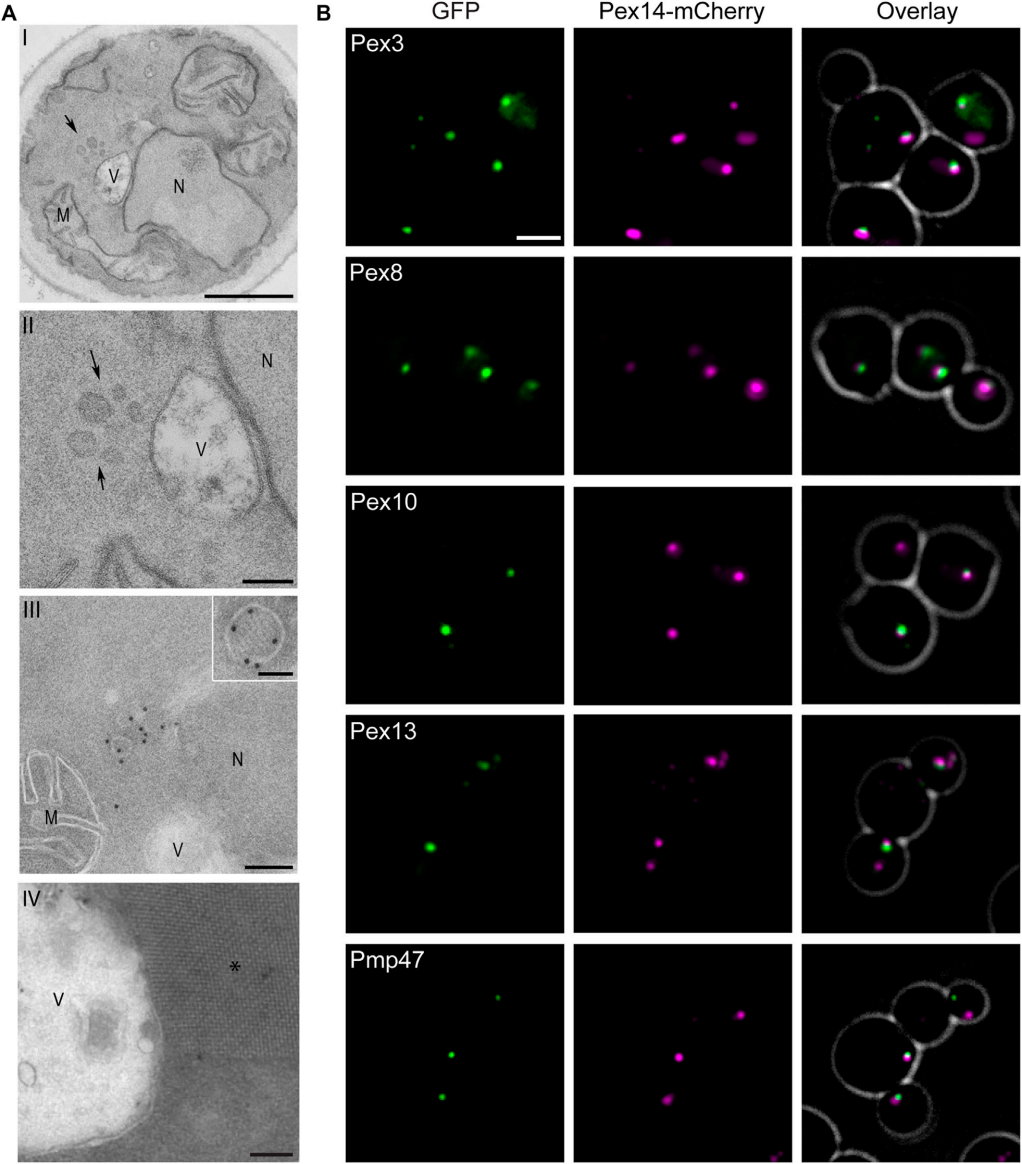
*H. polymorpha pex11 vps13* cells contain small peroxisomes. **(A)** Electron microscopy analysis of thin sections of KMnO_4_-fixed *pex11 vps13* cells grown for 8 h on a mixture of glycerol and methanol. Cells contain clusters of small peroxisomes (arrows). I—overview, II—magnification. III—Immunolabelling of cryosection of *pex11 vps13* cells using anti-Pex14 antibodies. The inset shows a small peroxisome labelled with anti-Pex14 antibodies, containing an alcohol oxidase crystalloid. IV—Cryosection showing a large, cytosolic alchol oxidase crystalloid. Scale bars: I: 500 nm, II: 100 nm, III 100 nm, inset: 50 nm, IV—100 nm. M-mitochondrion; N—nucleus, V-vacuole. **(B)** FM images of *pex11 vps13* cells producing Pex14-mCherry together with the indicated mGFP fusion proteins, all produced under control of the endogenous promoters. Cells were grown for 8 h on glycerol/methanol medium. Cells producing Pex10-GFP were grown for 4 h on glycerol/methanol. Scale bar: 2 µm.

Electron microscopy (EM) revealed that *H. polymorpha pex11 vps13* cells harbor clusters of very small peroxisomes (Figures 2AI,II), which contain the peroxisomal membrane marker Pex14 (Figure 2AIII). In the larger organelles small alcohol oxidase (AO) crystalloids could be observed, indicating that these peroxisomes still are capable of importing of matrix protein (Figure 2AIII, inset). The bulk of the AO protein mislocalizes to the cytosol, as evident from the large AO crystalloids in the cytosol of the double mutant (Figure 2AIV). Fluorescence microscopy showed that Pex14-mCherry localized in spots in *pex11 vps13* cells, which most likely represent the clusters of small peroxisomes observed by EM (Figure 2B). All other PMPs, C-terminally tagged with GFP and produced under the control of their endogenous promoters, co-localized with Pex14-mCherry (Figure 2B). Based on these data we conclude that PMPs normally localize to the membranes of the small peroxisomes in *pex11 vps13* cells. The small peroxisomes are competent to import a minor fraction of the matrix proteins, but the bulk of the matrix proteins mislocalizes to the cytosol.

### *VPS13* is also Required for Peroxisome Biogenesis in *H. polymorpha pex23* and *pex24* Cells

**FIGURE 3:**
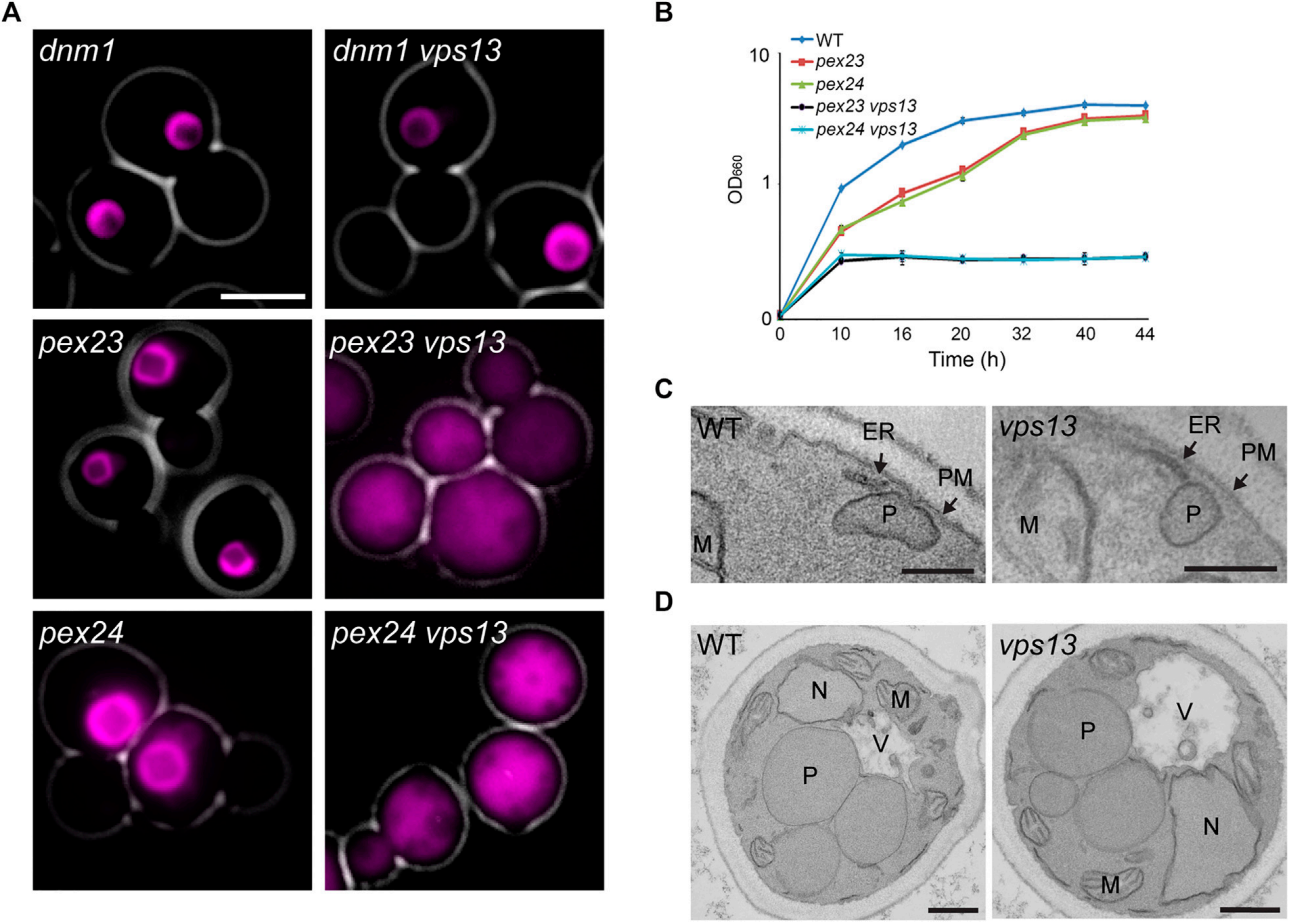
*H. polymorpha pex23 vps13* and *pex24 vps13* cells show a severe peroxisome biogenesis defect. **(A)** FM analysis of the indicated strains producing DsRed-SKL or GFP-SKL, grown for 16 h on a mixture of glycerol and methanol. Scale bar: 2 μm. **(B)** Growth curves of the indicated strains on medium containing methanol. The optical density is expressed as OD_660_. Error bars represent SD (*n* = 2). **(C,D)** Electron microscopy analysis of KMnO4 fixed WT and *vps13* cells grown on glucose **(C)** or methanol **(D)**. Like the WT controls *vps13* cells still contain peroxisome-ER, peroxisome-plasma membrane (PM), peroxisome-mitochondrion (M) and peroxisome-vacuole (V) contacts. Scale bar in C 200 nm, size bar in D 500 nm. N—nucleus.

Next, we analyzed whether deletion of *VPS13* also affects peroxisome biogenesis in *pex23* and *pex24* cells, two other mutants in which peroxisome-ER MCSs are reduced (Wu et al., 2020). As shown in Figure 3A, peroxisomal matrix markers mislocalized to the cytosol in *pex23 vps13* and *pex24 vps13* cells. In line with these observations, both double deletion strains are unable to grow on methanol, while *pex23* and *pex24* single deletion strains grow on methanol (Figure 3B).

The decrease in peroxisome numbers in *pex11*, *pex23* and *pex24* cells is related to reduced ER-peroxisome contacts (Wu et al., 2020). In *H. polymorpha dnm1* cells peroxisome abundance is also decreased, but not due to reduced contacts but caused by a block in peroxisome fission (Nagotu et al., 2008). Hence, we reasoned that deletion of *VPS13* in *dnm1* cells should not lead to enhanced peroxisome biogenesis defects. Indeed, as shown in Figure 3A, the peroxisome phenotypes of *dnm1* and *dnm1 vps13* cells are comparable. This observation supports the redundant function of Vps13 with peroxisome-ER contacts. Peroxisome-ER contacts still occur in *vps13* cells (Figure 3C). Also, in the absence of Vps13 all other peroxisomal contacts that were identified in *H. polymorpha* wild-type cells are normally present (peroxisome-plasmamembrane, Figure 3C; peroxisome-mitochondrion (M) and peroxisome-vacuole (V), Figure 3D).

### An Artificial Peroxisome-ER Tether Suppresses the *pex11 vps13*, *pex23 vps13* and *pex24 vps13* Phenotypes

**FIGURE 4:**
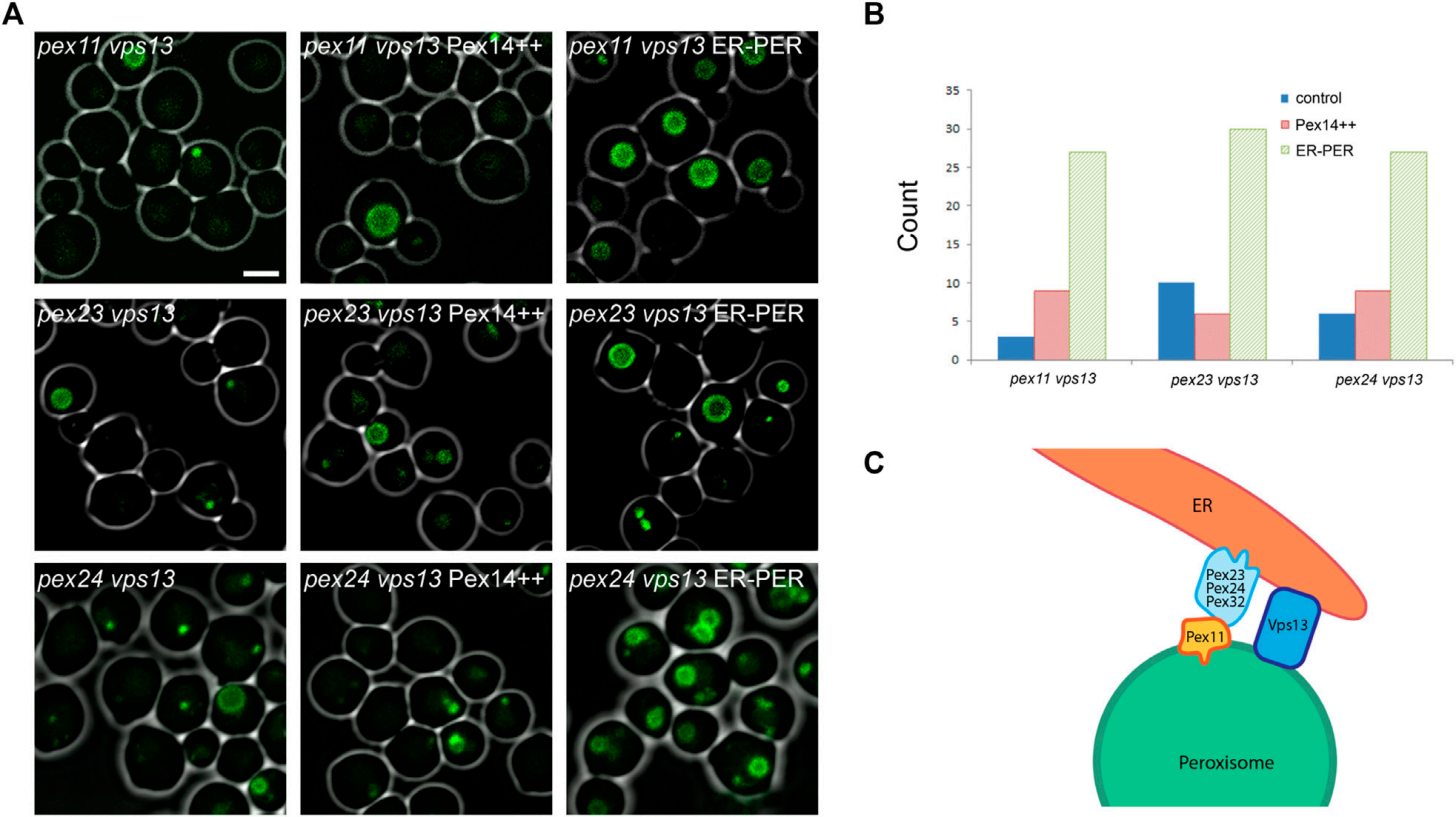
Suppression of peroxisome biogenesis defects by an artificial ER-peroxisome tether. **(A)** FM analysis of methanol-glycerol grown *pex11 vps13*, *pex23 vps13* and *pex24 vps13* cells producing P_*PMP47*_ *PMP47-GFP* alone or in combination with P_*ADH1*_ *PEX14* (Pex14++) or P_*ADH1*_ *PEX14-HAHA-UBC6* ^*TA*^ (ER-PER). Scale bar: 2 μm. **(B)** Quantitative analysis of the presence of peroxisomes in thin sections of methanol-glycerol grown cells of the indicated strains. Thin sections of KMnO_4_ fixed cells were analyzed by electron microscopy. The number of cell sections containing one or more peroxisomes with a diameter >200 nm were counted. 100 cell sections were analyzed per strain (*n* = 100). **(C)** Hypothetical model of the peroxisome-ER contact site in *H. polymorpha*, with the currently known components.

The peroxisomal defects that occur in *H. polymorpha pex23* and *pex24* cells are largely suppressed upon introduction of an artificial peroxisome-ER tether (Wu et al., 2020). This tether consists of full-length Pex14 and the tail anchor of the ER protein Ubc6, separated by two heme-agglutinin tags. Deletion of *VPS13* enhances the peroxisomal defects in *pex11, pex23* or *pex24* cells (Figure 1). Introduction of the artificial peroxisome-ER tether resulted in partical suppression of the severe peroxisomal phenotypes of the *pex11 vps13*, *pex23 vps13* and *pex24 vps13* double deletion strains (Figures 4A,B). This was not observed in control strains in which Pex14, which is part of the artificial tether, was overproduced (Pex14++; Figures 4A,B). These data suggest that upon artificially enhancing peroxisome-ER contacts again, Vps13 becomes less important for peroxisome biogenesis in the three indicated double deletion strains.

## Discussion

Here we identified *VPS13* as a gene required for peroxisome biogenesis in *H. polymorpha* cells with disturbed peroxisome-ER contacts. Previous data suggested that these contacts are involved in lipid transfer from the ER to peroxisomes to allow membrane expansion (Wu et al., 2020). This is in line with observations in *S. cerevisiae* indicating that the peroxisomal membrane can receive membrane lipids via non-vesicular transport (Raychaudhuri and Prinz, 2008) and that the ER is one of the lipid sources (Rosenberger et al., 2009; Flis et al., 2015).

Cells of the *H. polymorpha pex11, pex23* and *pex24* single deletion strains still contain peroxisomes, but show reduced growth on methanol media, due to a partial defect in peroxisome function (Wu et al., 2020). We now show that deletion of *VPS13* in each of these mutants results in a complete defect in methanol growth, accompanied by much more severe peroxisome biogenesis defects, including mislocalization of the bulk of the peroxisomal matrix enzymes in the cytosol (Figures 1E,F; Figures 3A,B). The latter is unlikely to be caused by defects in the importomer. First, peroxisomal membrane proteins, including proteins of the importomer, are still normally sorted to peroxisomes (Figure 2B). Second, peroxisomes still can incorporate a minor portion of the matrix proteins (Figure 2A). Third, the matrix protein import defect of the double mutants is largely restored by the introduction of an artificial ER-peroxisome tethering protein (Figure 4). Hence, the matrix protein import defect is most likely an indirect effect of the inability of the peroxisomes to sufficiently expand as a result of reduced membrane lipid supply.

Like for *H. polymorpha* also the *S. cerevisiae pex11 vps13* double mutant, but not the *pex11* and *vps13* single deletion strains, show severe peroxisome biogenesis defects, indicating that a role for Vps13 in peroxisome biogenesis is conserved in yeast (Figure 1G).

Vps13 was initially characterized as an *S. cerevisiae* protein involved in vacuolar protein sorting (Bankaitis et al., 1986). Later studies indicated that ScVps13 is required for many other processes, which all probably relate to a function in lipid transport. Vps13 is a very large (>300 kDa), highly conserved protein. In yeast, there is a single *VPS13* gene, while mammals contain four VPS13 isoforms: VPS13A-D. Protein structure analysis and biochemical studies revealed the presence of a large hydrophilic groove, which can bind and transfer a variety of glycerophospholipids. Most likely Vps13 is responsible for bulk transport of lipids between membranes, because its hydrophilic groove can bind multiple lipids at once (Leonzino et al., 2021).

*H. polymorpha* Vps13 is very similar to *S. cerevisiae* Vps13 (sequence identity 40%). Also, their length is similar (3135 and 3144 residues, respectively). Like the four human Vps13 homologues and *S. cerevisiae* Vps13, *H. polymorpha* Vps13 contains a chorein domain at the N-terminus and a VAB domain folllowed by an APT1, ATG2_C and plekstrin homology domain at the C-terminus (Dziurdzik and Conibear, 2021). *H. polymorpha* Vps13 has 23% identity with each of the four human Vps13 proteins.

*S. cerevisiae* Vps13 localizes to multiple MCSs, including the Nuclear Vacuolar Junction (NVJ), the vacuole-mitochondria patch (vCLAMP) and endosome-mitochondrial contact sites (Dziurdzik and Conibear, 2021). In addition, *S. cerevisiae* Vps13 localizes to prospore membranes (Park and Neiman, 2012), the Golgi apparatus (Kolakowski et al., 2021) and peroxisomes (John Peter et al., 2017). Most likely *H. polymorpha* Vps13 also localizes to multiple organelles. Human VPS13 isoforms also localize to different MCSs and cell organelles (Dziurdzik and Conibear, 2021). Moreover, recent studies showed that human VPS13D plays a role in peroxisome formation (Baldwin et al., 2021) and localizes to these organelles (Guillen-Samander et al., 2021).

VPS13D associates to both peroxisomes and mitochondria via Miro proteins that have a dual localization at both organelles. In addition, VPS13D associates to ER localized VAP proteins. In this way VPS13D connects the ER to peroxisomes and mitochondria to mediate bulk lipid transport (Guillen-Samander et al., 2021).

We generated a functional, internally GFP-tagged *H. polymorpha* Vps13 variant, but unfortunately the fluorescence levels were too low to determine its localization. However, given the peroxisomal localization of *S. cerevisiae* Vps13 and human VPS13D it is very likely that *H. polymorpha* Vps13 localizes to peroxisomes as well.

We were unable to detect a peroxisomal phenotype of *H. polymorpha* or *S. cerevisiae vps13* cells. Moreover, electron microscopy revealed that all described peroxisomal MCSs still occur in *H. polymorpha vps13* cells (Figure 3C). Similarly, Baldwin and others reported that *S. cerevisiae vps13* cells show no peroxisomal defects (Baldwin et al., 2021). This observation indicates that yeast Vps13 is redundant for peroxisome formation, but becomes important for lipid transport to peroxisomes, when the peroxisome-ER MCS formed by Pex23, Pex24, Pex32 and Pex11 are disturbed (Figure 4C). The defects were not only observed in methanol-grown *pex11 vps13* cells, but also when cells were grown on glucose (Figure 1C). In these cells peroxisomes mostly form contacts with the plasma-membrane and the ER (Wu et al., 2019). The contacts with the plasma membrane function in peroxisome retention (Krikken et al., 2020). Therefore Vps13 possibly performs its redundant function at peroxisome-ER contacts (Figure 4C). However, given the ambiguity of Vps13 function, other models cannot be excluded yet.

Further studies are required to understand the molecular mechanisms of Vps13 function in peroxisome formation. For instance, it would be important to know whether Vps13 plays a role in lipid transport, membrane tethering or both. This could be achieved by the introduction of mutations that affect lipid transfer in Vps13. Such mutations have been reported for *S. cerevisiae* Vps13 (Li et al., 2020). Hence, it would be interesting to learn whether corresponding mutations in *H. polymorpha* Vps13 abolish peroxisome formation in *pex11, pex23* and *pex24* cells. Our data indicate that *H. polymorpha* Vps13 functions together with Pex23 and Pex24 in peroxisomal membrane expansion. Like other protein of the Pex23 family, Pex23 and Pex24 contain a dysferlin (DysF) domain (Jansen et al., 2021). DysF was first identified in dysferlin, a human protein important for membrane repair of the sarcolemma at the site of muscle injury. The function of this domain is still unknown (Bulankina and Thoms, 2020). Interestingly, *S. cerevisiae* Spo73, another yeast DysF domain containing protein, also functions together with Vps13. Spo73 is required for extension of the prospore membrane and interacts with Spo71, which recruits Vps13 to the prospore membrane (Parodi et al., 2015; Okumura et al., 2016). It is tempting to speculate that members of the Pex23 family and Vps13 are components of a protein complex at peroxisome-ER contacts together with a yet unknown Vps13 recruiting protein at the peroxisomal membrane. Possibly the DysF domain is involved in the formation of this complex.

## Data Availability

The raw data supporting the conclusion of this article will be made available by the authors, without undue reservation.
