# Supplementary material for: Yeast Vps13 is Crucial for Peroxisome Expansion in Cells With Reduced Peroxisome-ER Contact Sites
Source: Front Cell Dev Biol. 2022 Feb 17;10:842285. doi: 10.3389/fcell.2022.842285 (PMC8891532; doi:10.3389/fcell.2022.842285)
Supplement: Supplementary file 1 [file DataSheet1.PDF]

Supplementary Material

1 Supplementary Tables

Supplementary Table 1. ***Hansenula polymorpha*** **strains used in this study**

**Strains**	**Description**	**Reference**
WT	NCYC495, *leu1.1*	(Sudbery et al, 1988)
yku80	NCYC495 *YKU80*::*URA3*	(Saraya et al, 2012)
WT *PMP47*-mGFP	pMCE7::*sh ble*	(Manivannan et al, 2013)
pex11 PMP47-mGFP	*PEX11*::*URA3* pMCE07::*sh ble*	(Thomas et al, 2015)
pex23 PMP47-mGFP	*PEX23*::*sh ble YKU80*::*URA3* pHIPN-*PMP47*-GFP::*NAT*	(Wu et al, 2020)
pex24 PMP47-mGFP	*PEX24*::*sh ble YKU80*::*URA3* pHIPN-*PMP47*-GFP::*NAT*	(Wu et al, 2020)
pex11	*PEX11*::*URA3*	(Krikken et al, 2009)
vps13	*VPS13*::*sh ble YKU80*::*URA3*	This study
pex11 vps13	*PEX11*::*URA3 VPS13*::*HPH*	This study
HF246 GFP-SKL	pHI-GFP-SKL::*LEU2*	(van Dijk et al, 2001)
vps13 GFP-SKL	*VPS13*::*sh ble* pHIPX7-GFP-SKL::*LEU2 YKU80*::*URA3*	This study

pex11 GFP-SKL	*PEX11*::*URA3* pHIPZ4-GFP-SKL::*sh ble*	(Nagotu et al, 2008)
pex11 vps13 GFP-SKL	*PEX11*::*URA3 VPS13::HPH* pHIPZ7-GFP-SKL::*sh ble*	This study
pex11 vps13 PEX14-mCherry *PEX3*-mGFP	*PEX11*::*URA3 VPS13*::*HPH* pHIPN-*PEX14*-mCherry::*NAT* pHIPZ-*PEX3*-GFP::*sh ble*	This study
pex11 vps13 PEX14-mCherry *PEX8*-mGFP	*PEX11*::*URA3 VPS13*::*HPH* pHIPN-*PEX14*-mCherry::*NAT* pMCE4::*sh ble*	This study
pex11 vps13 PEX14-mCherry *PEX10*-mGFP	*PEX11*::*URA3 VPS13*::*HPH* pHIPN-*PEX14*-mCherry::*NAT* pMCE5::*sh ble*	This study
pex11 vps13 PEX14-mCherry *PEX13*-mGFP	*PEX11*::*URA3 VPS13*::*HPH* pHIPN-*PEX14*-mCherry::*NAT* pSEM03::*sh ble*	This study
pex11 vps13 PEX14-mCherry *PMP47*-mGFP	*PEX11*::*URA3 VPS13*::*HPH* pHIPN-*PEX14*-mCherry::*NAT* pMCE7::*sh ble*	This study
dnm1 DsRed-SKL	*DNM1*::*LEU2* pHI-DsRed-SKL::*URA3*	(Cepinska et al, 2011)
dnm1 vps13 DsRed-SKL	*VPS13*::*sh ble DNM1*::*HPH* pHIPN4-DsRed-SKL::*NAT YKU80*::*URA3*	This study
pex23 DsRed-SKL	*PEX23::sh ble* pHIPN4-DsRed-SKL::*NAT YKU80*::*URA3*	This study
pex23 vps13.DsRed-SKL	*PEX23*::*sh ble VPS13*::*HPH* pHIPN4-DsRed-SKL::*NAT YKU80*::*URA3*	This study
pex24 GFP-SKL	*PEX24*::*sh ble YKU80*::*URA3* pHIPX4-GFP-SKL::*LEU2*	(Wu et al, 2020)

pex24 vps13 GFP-SKL	*PEX24*::*sh ble VPS13*::*HPH* *YKU80*::*URA3* pHIPX4-GFP-SKL::*LEU2*	This study
pex23	*PEX23*::*sh ble YKU80*::*URA3*	(Wu et al, 2020)
pex24	*PEX24*::*sh ble YKU80*::*URA3*	(Wu et al, 2020)
pex23 vps13	*PEX23*::*sh ble VPS13*::*HPH* *YKU80*::*URA3*	This study
pex24 vps13	*PEX23*::*sh ble VPS13*::*HPH* *YKU80*::*URA3*	This study
pex11 vps13 PMP47-GFP P_{ADH1}*PEX14*	*PEX11*::*URA3 VPS13*::*HPH* pHIPN-*PMP47*-GFP::*NAT* pARM059::*sh ble*	This study
pex11 vps13 PMP47-GFP P_{ADH1} *PEX14*-2xHA-*UBC6*	*PEX11*::*URA3 VPS13*::*HPH* pHIPN-*PMP47*-GFP::*NAT* pARM053::*sh ble*	This study
pex23 vps13 PMP47-GFP P_{ADH1}*PEX14*	*PEX23*::*sh ble VPS13*::*HPH* pHIPN-*PMP47*-GFP::*NAT* pARM069::*LEU2* *YKU80*::*URA3*	This study
pex23 vps13 PMP47-GFP P_{ADH1}*PEX14*-2xHA-*UBC6*	*PEX23*::*sh ble VPS13*::*HPH* pHIPN-*PMP47*-GFP::*NAT* pARM072::*LEU2* *YKU80*::*URA3*	This study
pex24 vps13 PMP47-GFP P_{ADH1}*PEX14*	*PEX24*::*sh ble VPS13*::*HPH* pHIPN-*PMP47*-GFP::*NAT* pARM069::*LEU2* *YKU80*::*URA3*	This study
pex24 vps13 PMP47-GFP P_{ADH1}*PEX14*-2xHA-*UBC6*	*PEX24*::*sh ble VPS13*::*HPH* pHIPN-*PMP47*-GFP::*NAT* pARM072::*LEU2* *YKU80*::*URA3*	This study

Supplementary Table 2. *S. cerevisiae* strains used in this study

WT	BY4742	Euroscarf collection
WT *GFP-SKL*	BY4742, pSL34::*sh ble*	This study
pex11 GFP-SKL	BY4742, *PEX11*::*KanMX*, pSL34::*sh ble*	This study
vps13 GFP-SKL	BY4742, *VPS13*::*KanMX*, pSL34::*sh ble*	This study
pex11 vps13 GFP-SKL	BY4742, *VPS13*::*KanMX PEX11*::*NAT*, pSL34::*sh ble*	This study

Supplementary Table 3. Plasmids used in this study

**Plasmids**	**Description**	**References**
pREMI-Z	REMI plasmid for transposon mutagenesis, Zeo^R, Amp^R	(van Dijk et al, 2001)
pMCE7	pHIPZ plasmid containing gene encoding C-terminal of Pmp47 fused to mGFP; Zeo^R, Amp^R	(Saraya et al, 2012)
pHIPN-*PMP47*-GFP	pHIPN plasmid containing C-terminal of Pmp47 fused to mGFP; Nat^R, Amp^R	This study
pHI-GFP-SKL	pHI plasmid containing GFP-SKL under the control of P_{AOX}; *URA3*, Amp^R	(Cepinska et al, 2011)
pHIPX7-GFP-SKL	pHIPX plasmid containing GFP-SKL under the control of P_{TEF}; *LEU2*, Kan^R	(Baerends et al, 1997)
pHIPZ4-GFP-SKL	pHIPZ plasmid containing GFP-SKL under the control of P_{AOX}; Zeo^R, Amp^R	(Leao-Helder et al, 2003)
pHIPZ7-GFP-SKL	pHIPZ plasmid containing GFP-SKL under the control of P_{TEF}; Zeo^R, Amp^R	(Knoops et al, 2014)
pSL34	Plasmid containing GFP-SKL under the control of P_{MET25}; Zeo^R, Amp^R	(Lefevre et al, 2013)
pSEM01	pHIPN plasmid containing gene encoding C-terminal part of Pex14 fused to mCherry; Nat^R, Amp^R	(Knoops et al, 2014)
pHIPZ-*PEX3*-mGFP	pHIPZ plasmid containing gene encoding C-terminal of Pex3 fused to mGFP; Zeo^R, Amp^R	This study

pMCE4	pHIPZ plasmid containing gene encoding C-terminal of Pex8 fused to mGFP; Zeo^R, Amp^R	(Cepinska et al, 2011)
pMCE5	pHIPZ plasmid containing gene encoding C-terminal of Pex10 fused to mGFP; Zeo^R, Amp^R	(Cepinska et al, 2011)
pSEM03	pHIPZ plasmid containing gene encoding C-terminal of Pex13 fused to mGFP; Zeo^R, Amp^R	(Knoops et al, 2014)
pMCE7	pHIPZ plasmid containing gene encoding C-terminal of Pmp47 fused to mGFP; Zeo^R, Amp^R	(Cepinska et al, 2011)
pHI-DsRed-SKL	pHI plasmid containing DsRed-SKL under the control of P_{AOX}; *URA3*, Amp^R	(Nagotu et al, 2008)
pHIPN4-DsRed-SKL	pHIPN plasmid containing DsRed-SKL under the control of P_{AOX}; Nat^R, Amp^R	(Cepinska et al, 2011)
pHIPX4-GFP-SKL	pHIPX plasmid containing GFP-SKL under the control of P_{AOX}; *LEU2*, Kan^R	(Faber et al, 2002)
pARM059	pHIPZ plasmid containing *PEX14* under the control of P_{ADH1}; Zeo^R, Amp^R	(Wu et al, 2020)
pARM053	pHIPZ plasmid containing *PEX14*-2xHA-*UBC6* under the control of P_{ADH1}; Zeo^R, Amp^R	(Wu et al, 2020)
pARM069	pHIPX plasmid containing *PEX14* under the control of P_{ADH1}; *LEU2*, Kan^R	This study
pARM072	pHIPX plasmid containing *PEX14*-2xHA-*UBC6* under the control of P_{ADH1}; *LEU2*, Kan^R	This study

Supplementary Table 4. Genes identified by transposon mutagenesis of *H. polymorpha pex11* cells

**Genes identified**	**Times found**	**Function**
PEX1	1	Matrix protein import
PEX2	2	Matrix protein import
PEX4	1	Matrix protein import
PEX5	3	Matrix protein import
PEX6	3	Matrix protein import
PEX8	4	Matrix protein import
PEX10	2	Matrix protein import
PEX12	3	Matrix protein import
PEX25	2	PMP with unknown function
PEX26	2	Matrix protein import
AMO	3	Amine oxidase
IRA1	1	GTPase activating protein
MUT3	1	Alcohol oxidase activation
HPODL_04236	1	Hypothetical protein
HPODL_05268	1	Putative transcription factor
MPP1	3	Transcription factor
VPS13	9	Involved in prospore membrane formation, localizes to several membrane contact sites;

References

Baerends RJ, Salomons FA, Faber KN, Kiel JA, Van der Klei IJ, Veenhuis M (1997) Deviant Pex3p levels affect normal peroxisome formation in Hansenula polymorpha: high steady-state levels of the protein fully abolish matrix protein import. *Yeast* **13:** 1437-1448

Cepinska MN, Veenhuis M, van der Klei IJ, Nagotu S (2011) Peroxisome fission is associated with reorganization of specific membrane proteins. *Traffic* **12:** 925-937

Faber KN, Haan GJ, Baerends RJ, Kram AM, Veenhuis M (2002) Normal peroxisome development from vesicles induced by truncated Hansenula polymorpha Pex3p. *The Journal of biological chemistry* **277:** 11026-11033

Knoops K, Manivannan S, Cepinska MN, Krikken AM, Kram AM, Veenhuis M, van der Klei IJ (2014) Preperoxisomal vesicles can form in the absence of Pex3. *The Journal of cell biology* **204:** 659-668

Krikken AM, Veenhuis M, van der Klei IJ (2009) Hansenula polymorpha pex11 cells are affected in peroxisome retention. *The FEBS journal* **276:** 1429-1439

Leao-Helder AN, Krikken AM, van der Klei IJ, Kiel JA, Veenhuis M (2003) Transcriptional down-regulation of peroxisome numbers affects selective peroxisome degradation in Hansenula polymorpha. *The Journal of biological chemistry* **278:** 40749-40756

Lefevre SD, van Roermund CW, Wanders RJ, Veenhuis M, van der Klei IJ (2013) The significance of peroxisome function in chronological aging of Saccharomyces cerevisiae. *Aging cell* **12:** 784-793

Manivannan S, de Boer R, Veenhuis M, van der Klei IJ (2013) Lumenal peroxisomal protein aggregates are removed by concerted fission and autophagy events. *Autophagy* **9:** 1044-1056

Nagotu S, Krikken AM, Otzen M, Kiel JA, Veenhuis M, van der Klei IJ (2008) Peroxisome fission in Hansenula polymorpha requires Mdv1 and Fis1, two proteins also involved in mitochondrial fission. *Traffic* **9:** 1471-1484

Saraya R, Krikken AM, Kiel JA, Baerends RJ, Veenhuis M, van der Klei IJ (2012) Novel genetic tools for Hansenula polymorpha. *FEMS yeast research* **12:** 271-278

Sudbery PE, Gleeson MA, Veale RA, Ledeboer AM, Zoetmulder MC (1988) Hansenula polymorpha as a novel yeast system for the expression of heterologous genes. *Biochemical Society transactions* **16:** 1081-1083

Thomas AS, Krikken AM, van der Klei IJ, Williams CP (2015) Phosphorylation of Pex11p does not regulate peroxisomal fission in the yeast Hansenula polymorpha. *Scientific reports* **5:** 11493

van Dijk R, Faber KN, Hammond AT, Glick BS, Veenhuis M, Kiel JA (2001) Tagging Hansenula polymorpha genes by random integration of linear DNA fragments (RALF). *Molecular genetics and genomics : MGG* **266:** 646-656

Wu F, de Boer R, Krikken AM, Aksit A, Bordin N, Devos DP, van der Klei IJ (2020) Pex24 and Pex32 are required to tether peroxisomes to the ER for organelle biogenesis, positioning and segregation in yeast. *Journal of cell science* **133**
